# Ontogeny of Toll-Like Receptor Mediated Cytokine Responses of Human Blood Mononuclear Cells

**DOI:** 10.1371/journal.pone.0015041

**Published:** 2010-11-30

**Authors:** Nathan P. Corbett, Darren Blimkie, Kevin C. Ho, Bing Cai, Darren P. Sutherland, Arlene Kallos, Juliet Crabtree, Annie Rein-Weston, Pascal M. Lavoie, Stuart E. Turvey, Natalie R. Hawkins, Steven G. Self, Christopher B. Wilson, Adeline M. Hajjar, Edgardo S. Fortuno, Tobias R. Kollmann

**Affiliations:** 1 Division of Infectious and Immunological Diseases, Department of Pediatrics, University of British Columbia, Vancouver, Canada; 2 Department of Immunology, University of Washington, Seattle, Washington, United States of America; 3 Fred Hutchinson Cancer Research Center, Seattle, Washington, United States of America; 4 Bill & Melinda Gates Foundation, Seattle, Washington, United States of America; United States of America

## Abstract

Newborns and young infants suffer increased infectious morbidity and mortality as compared to older children and adults. Morbidity and mortality due to infection are highest during the first weeks of life, decreasing over several years. Furthermore, most vaccines are not administered around birth, but over the first few years of life. A more complete understanding of the ontogeny of the immune system over the first years of life is thus urgently needed. Here, we applied the most comprehensive analysis focused on the innate immune response following TLR stimulation over the first 2 years of life in the largest such longitudinal cohort studied to-date (35 subjects). We found that innate TLR responses (i) known to support Th17 adaptive immune responses (IL-23, IL-6) peaked around birth and declined over the following 2 years only to increase again by adulthood; (ii) potentially supporting antiviral defense (IFN-α) reached adult level function by 1 year of age; (iii) known to support Th1 type immunity (IL-12p70, IFN-γ) slowly rose from a low at birth but remained far below adult responses even at 2 years of age; (iv) inducing IL-10 production steadily declined from a high around birth to adult levels by 1 or 2 years of age, and; (v) leading to production of TNF-α or IL-1β varied by stimuli. Our data contradict the notion of a linear progression from an ‘immature’ neonatal to a ‘mature’ adult pattern, but instead indicate the existence of qualitative and quantitative age-specific changes in innate immune reactivity in response to TLR stimulation.

## Introduction

Newborns and young infants have long been known to suffer higher infectious morbidity and mortality as compared to older children and adults; they also often display suboptimal immune responses to vaccination [1], [2]. Several factors appear to be responsible for this observation, including differences between neonatal and adult innate as well as adaptive immune function [1], [3], [4], [5], [6]. As innate instructs adaptive immunity, deciphering the role of innate immune function during early life is paramount to design effective interventions–such as vaccines–for this highly vulnerable age group.

Much of the published literature examining innate immunity has focused on pattern recognition receptors (PRR), such as Toll-like receptors (TLR), which are involved in the recognition of conserved microbial pathogen-associated molecular patterns (PAMP) (reviewed in [7]). Recognition of a specific PAMP by a specific TLR triggers events that result in the initiation of antigen uptake, processing, and presentation, as well as expression of co-stimulatory molecules and secretion of cytokine mediators. Together, these responses support immediate innate effector functions, such as phagocytosis, and also direct the ensuing adaptive immune response. It is important to note that innate PRRs are essential in initiating and orchestrating the immune response not just to infection, but—through recognition of adjuvants—also direct the quantity, quality, and longevity of the adaptive immune response to vaccination [7]. Centrally involved in these processes are antigen-presenting cells (APC). The major human APCs are dendritic cells (DCs), both classical (cDC) and plasmacytoid (pDC), as well as monocytes/macrophages and B cells. Interestingly, each of these APC expresses a somewhat unique set of PRRs suggesting each cell type may have specific functions [7]. We thus conducted our analysis of innate responses to TLR stimulation in all four APC types in parallel and in the same sample.

Our previous studies and those of other groups had identified significant differences between cord and adult peripheral blood responses to TLR stimulation (reviewed in [1], [8]). Our comprehensive comparison of neonatal to adult innate response to TLR stimulation consisted of simultaneous analysis of both global (through cytokine quantification in the culture supernatant) as well as single-cell analysis (via intracellular cytokine staining (ICS)) of the response of all four major APCs to a broad range of well-defined TLR stimuli [9], [10], [11]. Using this platform, we had described that monocytes, cDC and pDC from neonates display a consistently and significantly reduced capacity compared to adults to produce IL-12p70, IFN-α2 and IFN-γ, with a more modest and less consistently reduced capacity to produce TNF-α, a similar or even greater capacity to produce IL-6, IL-23 and IL-1β, and a much greater capacity to produce IL-10 in response to TLR stimulation. These findings indicate that the neonatal innate immune system is in fact not less capable than that of the adult to respond to TLR stimulation, but instead responds in a qualitatively different manner. These differences may relate to the observed increased neonatal infectious morbidity and mortality as well as the suboptimal immune responses to vaccines administered around birth [1], [2], [8].

However, it is well recognized that while the incidence of infections is highest in the neonatal period, infants continue to remain at increased risk for several years [12], [13]. Furthermore, most vaccines are not administered around birth, but in infancy [2]. Therefore a more complete understanding of the ontogeny of the innate immune system over the first few years of life, i.e. beyond the neonatal period, is needed. In the current study, we have set out to apply our comprehensive approach to innate immune profiling following TLR stimulation over the first 2 years of life. We were able to do so following a cohort large enough to allow well-powered statistical analyses. Our findings contradict the notion of a linear progression from an ‘immature’ neonatal to a ‘mature’ adult pattern [14], but instead indicate the existence of qualitative and quantitative age-specific patterns in innate immune reactivity in response to TLR stimulation.

## Results

### Single-cell analysis of intracellular cytokine production by neonatal, one- and two-year old as well as adult innate mononuclear cells

Our flow cytometric platform for the analysis of TLR responses at the single-cell level had been described previously [9], [11] and has since been applied successfully comparing neonatal to adult responses to TLR stimulation [10]. This polychromatic single-cell high-throughput flow cytometric profiling approach allowed us to determine the cellular composition of the innate immune system in all mononuclear cells (MC) of interest (Figure S1). Contrasting the innate immune cell subset composition in MC from birth over the first 2 years of life to an adult group, we detected several significant changes in the relative percentage of the various APC amongst live MC (Figure S2). Specifically, the percentage of monocytes was found to be higher in cord blood and adult peripheral MC as compared to peripheral MC from 1- and 2-year olds, respectively. The opposite appeared to be the case for B cells; i.e. percentage of B cells was higher in MC from 1- and 2-year olds compared to MC from cord or adult blood, respectively. cDC increased from a low at birth to adult levels by 1-year of age. Finally, pDC remained lower than adult up to 2-years of age. These changes in cellular composition have to be kept in mind when interpreting data from, e.g., the culture supernatant multiplex analysis.

The ability to identify all four major human APC subsets in the same sample simultaneously provided the unique opportunity to assess the production of TNF-α, IL-6, IL-12/23p40, as well as IFN-α2 at the single-cell level in response TLR stimulation in all four subsets (Figure S3). As our overall analysis focused on the direct comparison of the longitudinal early-life samples to each other and adult MC samples after TLR-specific stimulation, we positioned each unstimulated flow cytometric sample as the biological negative control for each subject at the specific time point investigated; thus all unstimulated samples are associated with ∼0% cytokine-positive cells. This has been identified as the most appropriate approach for flow cytometric analysis of stimulation experiments such as ours [15].

**Figure 1:**
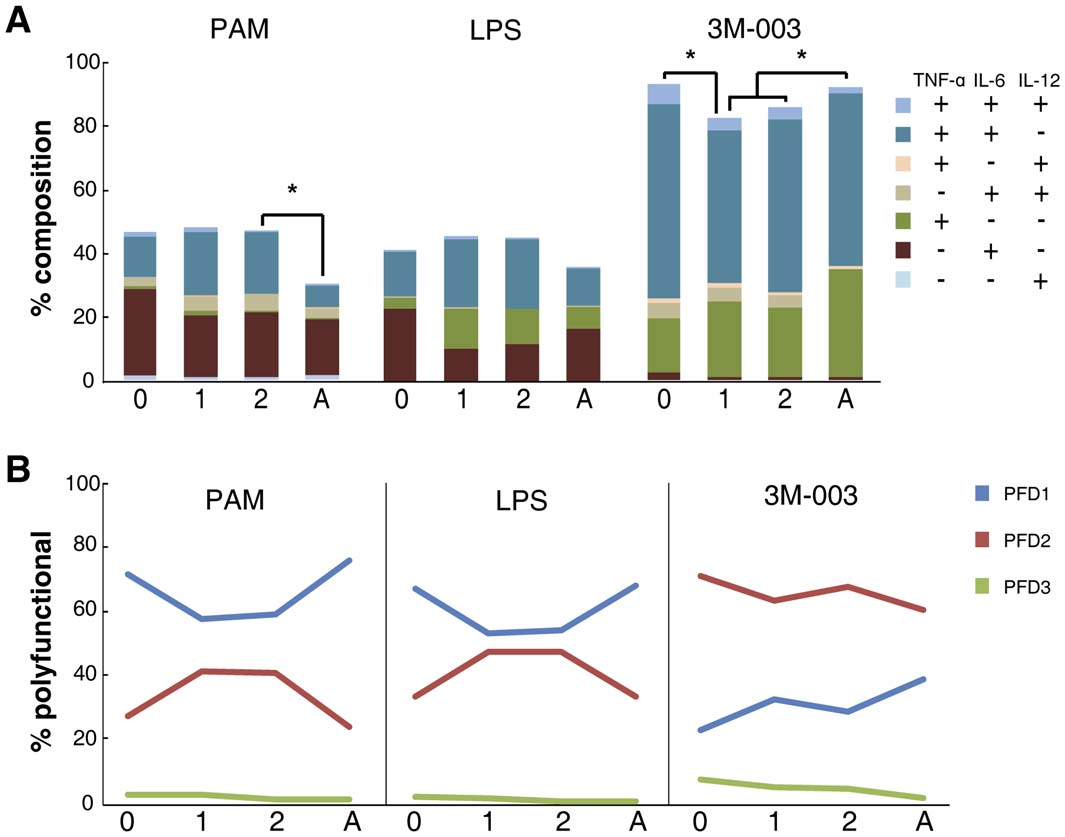
Longitudinal TLR response of monocytes as measured by intracellular cytokine staining. MC samples from 35 neonate-infant subjects (followed longitudinally as a cohort from birth (0), and then at 1-year (1) and 2-years (2) of life) and 25 adults (A) were stimulated with the indicated TLR ligands for 6 h, and the intracellular expression TNF-α, IL-6 and IL-12/23p40 by monocytes was determined by multi-parameter flow cytometry. (A) Stacked bar graphs in which the overall height of the bar indicates the total percentage of monocytes producing any cytokine (with statistically significant differences (p<0.01) between age groups indicated by *), and the height of each color indicates the percentage of monocytes expressing an individual cytokine or cytokine combination (coded as shown in the inset, arranged from top to bottom in the same order as the stacked bar). (B) The line graphs represent the qualitative summary of the polyfunctionality degree (PFD) for monocytes after TLR stimulation of MC from samples isolated at the different age groups.

**Figure 2:**
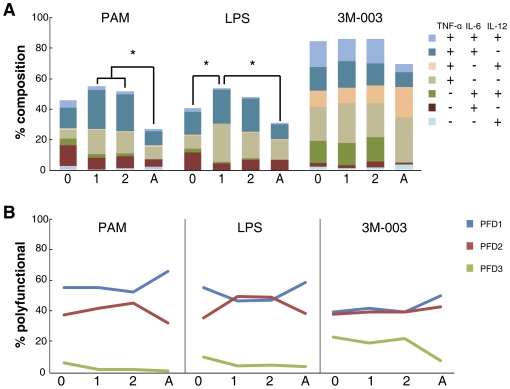
Longitudinal TLR response of cDC as measured by intracellular cytokine staining. MC samples from 35 neonate-infant subjects (followed longitudinally as a cohort from birth (0), and then at 1-year (1) and 2-years (2) of life) and 25 adults (A) were stimulated with the indicated TLR ligands for 6 h, and the intracellular expression TNF-α, IL-6 and IL-12/23p40 by cDC was determined by multi-parameter flow cytometry. (A) Stacked bar graphs in which the overall height of the bar indicates the total percentage of cDC producing any cytokine (with statistically significant differences (p<0.01) between age groups indicated by *), and the height of each color indicates the percentage of cDC expressing an individual cytokine or cytokine combination (coded as shown in the inset, arranged from top to bottom in parallel the stacked bar). (B) The line graphs represent the qualitative summary of the polyfunctionality degree (PFD) for cDC after TLR stimulation of MC from samples isolated at the different age groups.

**Figure 3:**
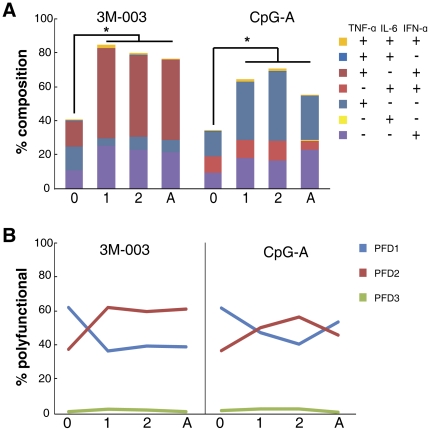
Longitudinal TLR response of pDC as measured by intracellular cytokine staining. MC samples from 35 neonate-infant subjects (followed longitudinally as a cohort from birth (0), and then at 1-year (1) and 2-years (2) of age) and 25 adults (A) were stimulated with the indicated TLR ligands for 6 h, and the intracellular expression TNF-α, IL-6 and IFN-α by pDC was determined by multi-parameter flow cytometry. (A) Stacked bar graphs in which the overall height of the bar indicates the total percentage of pDC producing any cytokine (with statistically significant differences (p<0.01) between age groups indicated by *), and the height of each color indicates the percentage of pDC expressing an individual cytokine or cytokine combination (coded as shown in the inset, arranged from top to bottom in parallel to the stacked bar). (B) The line graphs represent the qualitative summary of the polyfunctionality degree (PFD) for pDC after TLR stimulation of MC from samples isolated at the different age groups.

We determined previously that responses of both neonatal and adult samples were most pronounced at the highest TLR ligand concentration tested [10], [11], and thus have conducted the present study using only the highest effective yet non-toxic concentration. In our hands, the TLR9 ligand CpG-A did not induce a measurable response in either neonatal or adult cDC or monocytes; TLR8 stimulation did not provide any insight not gathered from TLR4 or TLR7/8 response analysis for monocytes and cDC, and TLR7 stimulation did not provide any insight not gathered from TLR7/8 or TLR9 analysis for pDC [10], [11]. Accordingly, we focused our current analysis on TLR2/1 (PAM_3_CSK_4_), TLR4 (LPS), and TLR7/8 (3M-003) for monocytes and cDC (Figures. 1 and 2), and TLR7/8 (3M-003) and TLR9 (CpG-A) stimulation for pDC (Figure 3). We and others have previously shown that soluble or other cellular factors contained in whole blood (WB) strongly affect the responses to TLR stimulation [8], [10]. For this study we wished to concentrate on the IL-12 and type 1 interferon-family responses, which, along with 3M-003 (TLR7/8), were consistently strongest with pI:C (TLR3) and CpG-A (TLR9) stimulation of PBMC as compared to WB. Thus, we decided to conduct this longitudinal innate immune profiling on MC instead of WB. Figure S3 shows an example of the response to TLR7/8 stimulation of monocytes, B cells, cDC as well as pDC from MC purified from cord blood, and peripheral blood from 1- and 2-year olds, and adults measured by flow cytometry. As we had previously shown for adult B cells [11], B cells from MC from neonates as well as from 1- and 2-year olds also failed to produce any of the 4 cytokines analyzed in response to any of the TLR stimuli we tested. Similar to what we had previously found [10], monocytes and cDC responded by making TNF-α, IL-6, and IL-12/23p40, but not IFN-α2, while pDC responded by making TNF-α, IL-6, and IFN-α2, but not IL-12/23p40. We thus show here our analysis only for each of these 3 cytokines in each respective subset. The percentages of cells producing one or any of the possible combinations of the cytokines tested can be depicted in color-coded stacked bar graphs such as seen in Figures1, 2, 3, where the total height of the bar corresponds to the total response (i.e., the percentage of cells producing any cytokine), and each colored segment corresponds to the percentage of cells producing a specific cytokine or combination of cytokines. In addition, to provide a more intuitive means to visualize the degree with which a given cell population responded to TLR stimulation by producing either any 1, any 2 or any 3 cytokines, we depicted the degree of polyfunctionality (PFD) of cells below the stacked bar graphs in Figures 1, 2, 3.

### Differences in monocytes and cDC cytokine production within early life and as compared to the adult

**Figure 4:**
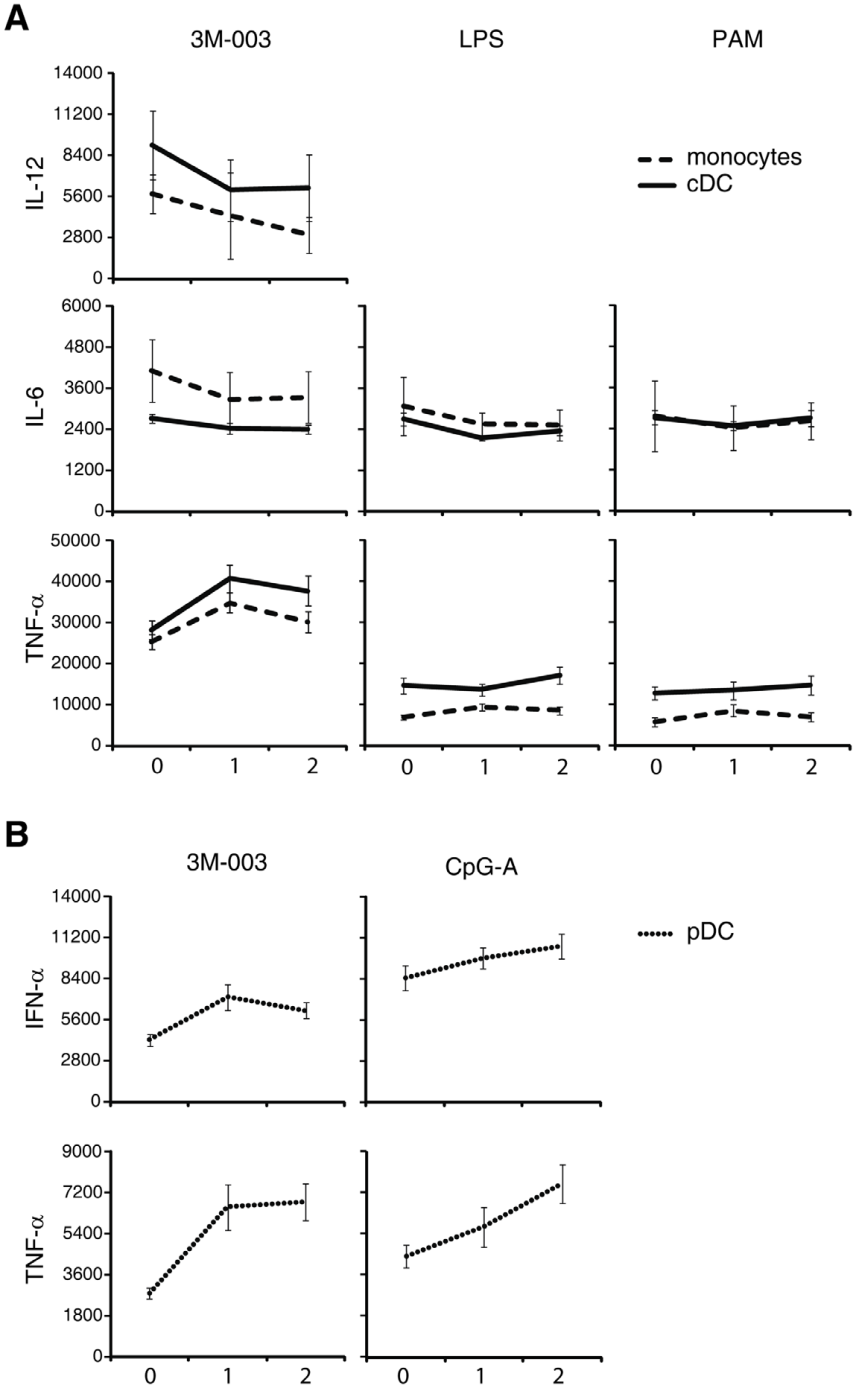
Mean fluorescence intensities of the different APC subpopulations that produced cytokines in response to TLR stimulation. The MFI values from samples ran by the same operator and on the same machine for 10 subjects over the first two years of life (0, 1, and 2 years of age) were averaged after first excluding the samples that have cytokine-positive percentage <4% of the APC subtype. Means for each population are derived from the FloJo software; error bars indicate SEM.

We found that while the total response (height of the stacked bar) of monocytes was no different between cord blood and peripheral blood of the same individuals at 1 and 2 years of age after TLR2/1 and TLR4 stimulation, all of the early-life age groups displayed a higher total response to PAM than adult samples; this however only reached statistical significance (p<0.01) in the comparison of 2 year old vs. adult (Figure 1A and Table S1). This contrasted with the response following TLR7/8 stimulation, where both neonate and adult monocytes displayed a significantly higher total response than monocytes from 1- or 2-year old subjects, respectively. The observed higher percentage of IL-6 producing monocytes after TLR2/1 stimulation in neonatal MC compared to adult was sustained up to the second year of life. There was no difference in the percentage of LPS induced IL-6 producing monocytes between any of the age groups. However TLR7/8 stimulation induced a higher percentage of IL-6 producing cells in cord blood as compared to the other age groups. IL-12/23p40 production was generally low in monocytes in all age groups, except after TLR7/8 stimulation, where cord blood contained a higher percentage of IL-12/23p40-positive monocytes as compared any other age group tested. TNF-α production appeared to differ between age groups and TLR stimuli. Adult blood had the lowest percentage of monocytes producing TNF-α in response to TLR2/1, but highest in response to TLR7/8 stimulation, while the fraction of monocytes producing TNF-α in response to TLR4 or TLR2/1 stimulation was higher in both 1- and 2-year old infant samples as compared with either neonatal or adult samples. The amount of cytokine produce per cell, i.e., the mean fluorescence intensity (MFI; see Figure 4A and Table S2), of monocytes for IL-6 did not change significantly from birth up to age 2 years for any of the TLR stimuli we tested. The MFI for TNF-α in monocytes following 3M-003 however increased from birth to 1 year and or 2 years of age. Lastly, while the fraction of monocytes able to produce more than 1 cytokine—i.e., their PFD2 and PFD3—decreased from birth to 1 and 2 years of life to adult following TLR7/8 stimulation, neonatal and adult samples appeared more similar as compared to infant samples following TLR2/1 or TLR4 stimulation, in that both cord and adult peripheral blood monocytes had a lower PFD2 as compared to 1- and 2-year old infant peripheral blood samples (Figure 1B).

We detected a higher total fraction (height of the stacked bar) of infant cDC as compared to adult cDC expressing cytokines in response to PAM, and higher total responses in the 1 year old as compared to either neonatal or adult cDC in response to LPS. Contrary to monocytes, there was no statistically significant difference in the response of cDC between the age groups following 3M-003 stimulation (Figure 2A; Table S1). The higher response in the 1- and 2-year infant samples as compared to neonatal or adult samples after TLR2/1 or TLR4 stimulation was largely due to a higher fraction of cells producing TNF-α alone or TNF-α with IL-6 in the infant cDC samples. The fraction of cDC producing IL-12/23p40 was highest in the cord blood sample after TLR7/8, but statistically significantly higher as compared to adult or infant only after PAM or LPS stimulation. The percentage of cDC producing IL-6 was higher in all early life age groups as compared to adults for any TLR ligand tested. In contrast, the percentage of cDC producing TNF-α was higher in the 1- and 2-year old infant samples as compared to either neonate or adult following TLR2/1 and TLR4 stimulation. There was no statistically significant difference between any of the age groups in the amount of IL-12/23p40 produced per cell (i.e., MFI); however, cord blood cDC produced more IL-6 as compared to infant cDC in response to LPS, but less TNF-α in response to 3M-003 (Figure 4A and Table S2). The fraction of cDC able to make 3 of the cytokines tested decreased from birth for all TLR stimuli (Figure 2B); however, while the fraction of cDC making 2 cytokines increased in infancy, we found it to be decreased in adults, such that adult cDC overall had the lowest polyfunctionality of all age groups tested.

### pDCs reach or exceed an adult-level response to TLR stimulation by 1-year of age

We confirmed our previous finding that neonatal pDCs were significantly less responsive as compared to adult pDC after either TLR7/8 or TLR9 stimulation (Figures 3A, 4B, and Tables S1 and S2). However, this difference disappeared by 1-year of age, as pDC from individuals at 1- and at 2-years of age had a total response at or above adult levels. This was the case for both IFN-α2 and TNF-α with respect to percentage of cells responding and the MFI of the responders after TLR7/8 or TLR9 stimulation (neither TLR2/1 nor TLR4 induced a significant response above background in pDC of any age group). Given that pDCs produced IFN-α, TNF-α or both, but little or no IL-6 or IL-12/23p40, pDCs were found to be at most bifunctional and not polyfunctional (Figure 3B). In response to TLR7/8 stimulation, there was a consistent increase in the fraction of bifunctional pDC after birth. There also was in increase in PFD after TLR9 stimulation; however, between age 2 years and adulthood, the fraction of bifunctional pDC after TLR9 stimulation decreased again.

### Global analysis of secreted cytokine production in response to TLR stimulation of neonatal, 1- and 2-year old, and adult MC cells

**Figure 5:**
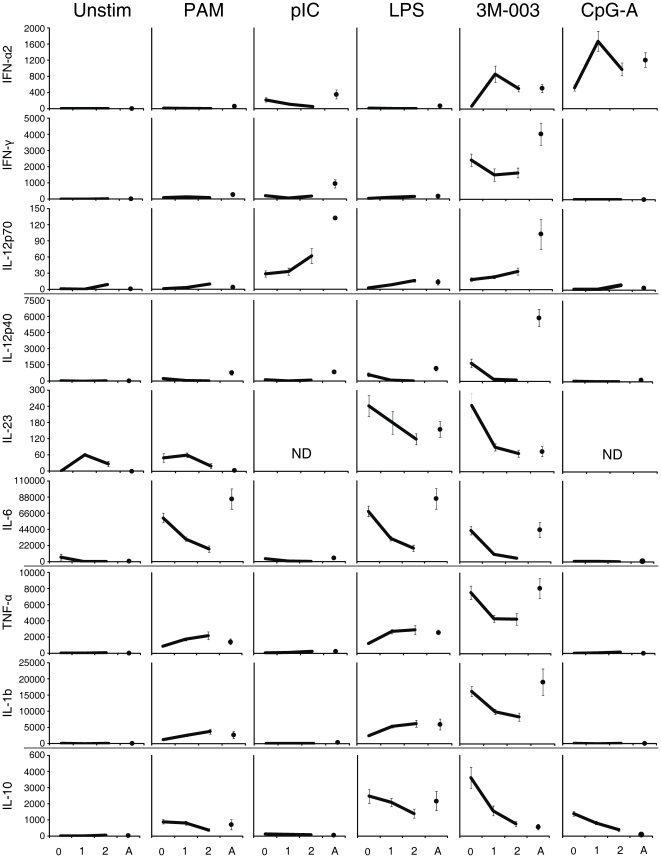
Bulk measurement of secreted cytokine production in response to TLR stimulation of MC from neonatal, 1- and 2-year olds, and adult. Cytokines secreted by MC in tissue culture containing the indicated TLR ligands were measured by ELISA (IL-23) or by Luminex's xMAP cytokine assays. The MC were stimulated for 18 h at 37°C, 5% CO_2_, after which the supernatants were harvested and frozen at −80°C until time of assay. The IL-23 sandwich ELISA was done on samples from 25 neonate-infant subjects (0, 1, 2) and 25 adult subjects (A). The other cytokines were assayed via Luminex multiplex analysis for 35 neonate-infant subjects and 25 adult subjects. The y-axes represent the mean cytokine concentration in pg/ml; error bars indicate SEM. ND  =  not done.

Our experiments were set up to also provide a global view of differences between neonatal and adult cells of cytokines produced after 18 h of stimulation by all cells present in the culture (Figure 5 and Table S3). Although less specific, i.e. the source of the cell type producing a given cytokine is not directly identifiable, this multiplexed bead array based approach offers the advantage of permitting quantitative analysis of cytokine responses for which there are no sufficiently sensitive or specific tools available for flow cytometric analysis (e.g. IL-12p70, IL-23). It also allows for cytokine networks to functionally interact *in vitro*, as cytokine secretion is not inhibited through brefeldin A (BFA). As we had previously shown, multiplexed bead array-based analysis and ICS do not always produce congruent results, but instead support and complement each other, in combination providing the currently most comprehensive approach to innate immune profiling [16].

#### TLR7/8 and TLR9 driven antiviral IFN-α2 responses reached adult levels by 1 year of life

As we had previously shown, following TLR7/8 (3M-003) or TLR9 (CpG-A) stimulation, production of IFN-α2 (the only IFN-α detected by our Luminex assay) was much reduced at birth as compared to adults. The IFN-α2 response to pI:C, although overall much lower as compared to TLR7/8 or TLR9 stimulation, was found to be similar between neonatal cord blood and adult peripheral blood. However, in response to either TLR7/8 or TLR9 stimulation, IFN-α2 appeared to be produced at levels similar to adult MC by 1 year of age, and this high-level response was maintained at least up to 2 years of age. In contrast, the IFN-α2 response to TLR3 stimulation actually decreased from birth to 1 and further by 2 years of age. Neither PAM nor LPS induced significant IFN-α2 production at any age.

#### Th-1 supporting IL-12p70 production increased but remained below adult levels even at 2 years of age

The production of IL-12p70 was strongest in all age groups in response to pI:C and 3M-003, with only marginal responses induced by LPS, and no significant response to PAM or CpG-A. The capacity to produce IL-12p70 displayed a steady increase from birth onwards, in that the same subjects produced more IL-12p70 at 1 year of age than at birth, and again more at 2 than at 1 year of age. But even at 2 years of age, the cohort we followed produced less than half of the amount of IL-12p70 as compared to our adult control group. This trend was the same for TLR3, TLR4, or TLR7/8 stimulation.

#### Th-1 supporting IFN-γ production always remained below adult levels, and in fact was found to decrease from birth up to age 2 years

IFN-γ production was only detectable after TLR7/8 stimulation. In contrast to the trend for IL-12p70, IFN-γ was observed to decrease after TLR7/8 stimulation between birth and 1 year of age, with no significant change thereafter. Importantly, at all early-life time points tested, IFN-γ production in response to TLR7/8 stimulation was found to be far below that of adult MC.

#### Th17-supporting IL-12p40, IL-23 and IL-6 cytokine production declined from birth over the first 2 years of life

We previously described a higher than adult-level of IL-23 production in cord blood MC. In response to LPS and 3M-003, we now found production of IL-23 to drop from the cord blood-high to adult-levels by 1-year of age, where it remained up until at least 2 years of age. This trend, a decline from birth to lower levels by 1 year of age was also observed for the IL-12/23p40 subunit, that together with the p19 subunit make up IL-23. However, contrary to IL-23, IL-12p40 in response to TLR4 or TLR7/8 stimulation was detected at much higher levels in the adult. It is important to remember that IL-12p40 also functions as a homodimer [17]. PAM did not induce production of IL-23 or IL-12p40 above background; however, PAM induced significant IL-6 production in all age groups, as did LPS and 3M-003. Similar to IL-12p40, IL-6 dropped from a high cord blood-level that was either at (for 3M-003) or just below (for PAM, LPS) the adult-level of production, to a lower level at 1-year of age and lower again at 2-years of age. As we had previously described [9], CpG-A and pI:C produced artifactual high reads for IL-23, and were thus not included in the IL-23 analysis. Neither pI:C nor CpG-A induced levels above unstimulated samples for either IL-12p40 or IL-6.

#### TNF-α and IL-1β both increased over the first 2 years of life for TLR2/1 as well as TLR4 responses, but not for TLR7/8 responses

We had previously described an adult-level production of TNF-α in cord blood in response to TLR7/8 stimulation, and observe the same here. The TNF-α response to 3M-003, however, dropped from the high in neonates to significantly lower levels by 1-year of age, where they were maintained up to at least age 2 years. IL-1β production in response to TLR7/8 stimulation followed the exact same pattern. However, contrary to the trend following TLR7/8 stimulation, both TNF-α and IL-1β increased from a level below the adult at birth to an adult-level production following TLR2/1 or TLR4 stimulation by 1 year of age, where it was maintained up until at least 2 years of age. Neither pI:C nor CpG-A induced production of either TNF-α or IL-1β above background in any of the age groups tested.

#### Anti-inflammatory IL-10 production declined from birth onwards to at or below adult- levels

Cord blood production of the anti-inflammatory and immune-regulatory cytokine IL-10 after TLR stimulation has previously been described by us and others as at or above adult levels. This was indeed the case for this cohort as well, in that TLR2/1 and TLR4 induced production of IL-10 in cord blood at levels similar to adult, and TLR7/8 and TLR9 stimulation induced production of IL-10 in cord blood at levels far above those found in adult MC. However, we found in this longitudinal study that, while the ability to produce IL-10 following TLR2/1 stimulation was maintained at adult levels up until the age of 1 year, it dropped to below neonatal and adult levels by 2 years of age. The IL-10 response following TLR4 stimulation declined from birth to two years of age. And lastly, IL-10 production following both TLR7/8 and TLR9 stimulation dropped from the high at birth by 1 year, and continued to drop to reach the low adult levels only by age 2 years. pI:C was not found to induce IL-10 production above background in any of the age groups tested.

## Discussion

Our study aimed to profile the human innate immune response to wide range of well-defined TLR ligands over the first 2 years of life. Using a well-established, robust, high-throughput profiling platform, we were able to follow a large number of children from birth to two years of age. We detected a pattern that suggests the existence of age-specific responses rather than a global, linear progression from a neonatal to an adult pattern. Specifically, we found an increase in the production of IFN-α2 that reached adult response levels after stimulation with TLR7/8 or TLR9 ligands by 1-year of age. This was also confirmed at the single-cell level, in that pDC acquired adult-like cytokine response patterns, including degree of polyfunctionality, by 1 year of age. This stood in contrast to the infants' capacity to produce IL-12p70, which in response to TLR3 and TLR7/8 increased from birth to 1 and again to 2 years of age, but even then never reached adult levels. And while the ability to produce TNF-α or IL-1β reached adult levels after TLR2/1 and TLR4 stimulation, it in fact decreased following stimulation with TLR7/8 to below adult levels by 1 year of age. IL-6, IL-12p40, IL-23 and IL-10 following TLR2/1, TLR4, and TLR7/8 (and TLR9 for IL-10) stimulation decreased by 1 year of age from the neonatal high level, and continued to decrease to either reach or even drop below adult levels of response. These findings suggest potentially important time periods during which vaccination may result in biased immune responses, or particular windows of vulnerability to specific pathogens. Deciphering the mechanisms underlying our observations is likely to yield important insights, and constitutes the necessary next step investigating innate immune ontogeny.

Most studies examining early life innate immune function have focused on cord blood [1], [8]. Only few previous studies have analyzed postnatal innate immune development [14], [18], [19], [20], [21]. Our study design offered several unique advantages over these previous studies. For example, ours was the only study that examined the ontogeny of innate immune responses in a truly longitudinal fashion–i.e. following the same subjects over time–this likely reduces the variability due to genetic heterogeneity [22]. Furthermore, we were able to enroll, retain and analyze high numbers of our subjects at each time point for each assay, allowing well-powered statistical analysis. Our study also was the first to analyze the postnatal ontogeny of the innate immune system using ICS in all 4 major APC subsets at the same time in the same sample, and the first to interrogate a wide-range of specific TLR responses using the currently most comprehensive innate profiling platform by coupling flow cytometry with a multiplexed bead array approach. Lastly, our cohort was followed over the same time period that the elevated risk for infectious diseases related morbidity and mortality has repeatedly been observed—the first 2 years of life [12]. The same time period also covers the span that most childhood vaccines are given [2].

Our analysis of the cellular composition of the human APC compartment in MC revealed several significant changes over the first 2 years of life, namely the juxtaposed increase of B cells and decrease of monocytes from birth to 1- and 2-years of age [23], [24]. We also observed a steady increase in cDC no increase in pDC over the first 2 years of life. Nguyen *et al.* and Belderbos *et al.* had looked at WB [18], [19], while we analyzed the innate cell content in MC. WB contains neutrophils, the predominant white blood cell in peripheral blood; neutrophil numbers are known to drop from a high in early life [23] leading to a potential relative increase in monocytes, cDC and pDC, precisely as described by Nguyen *et al.* and Belderbos *et al.* [18], [19]. The changes in the composition of APC in MC over the first few years of life we describe here may impact the cytokine quantification in bulk culture supernatant as shown in Figure 5.

Nguyen *et al.* followed children from birth up to only 1-year of age by analyzing the impact of TLR4 and TLR9 stimulation on APC surface maturation and cytokine secretion in WB cultures [18]. With respect to the 2 TLRs examined by Nguyen *et al.*, our findings are in full agreement with theirs, but importantly, our studies extend them to 2 years of age and include several additional qualitatively and quantitatively important insights. For example, similar to us, Nguyen *et al.* describe that the TNF-α response to LPS reaches adult levels by 1-year of age, and that the LPS induced IL-6 production drops from a high at birth to lower levels at 1-year of age. In our study, we extended this observation to show that this drop in IL-6 production in response to LPS continued up until at least 2-years of age, in fact to a level below that of the adult. Similarly to our results, Nguyen *et al.* also found no striking change in the very high early-life LPS induced IL-10 production by 1-year of age. Again, our findings extended this through our discovery of a pronounced drop in LPS induced IL-10 production to occur between 1- and 2-years of age to a level significantly below that of adults. Similar to our findings, Nguyen *et al.* detect a significant drop in CpG-A induced IL-6 and IL-10 production between birth and 1-year of age; again, we observed this drop to continue further between years 1 and 2 of life, and to also occur after TLR7/8 stimulation. Both our results and those of Nguyen *et al.* indicate that the early-life IL-12p70 response to LPS reaches adult-levels by 1-year of age. However, we found that even then the amount of IL-12p70 produced in response to LPS was still markedly lower as compared to, e.g., pI:C or 3M-003. Finally, for the high IL-12p70-inducing TLRs (TLR3 and TLR7/8), while production of IL-12p70 increased after birth, it remained at a significantly lower level than adults even up to 2-years of age.

The fact that our data and that of Nguyen *et al.* support each other up to at least 1 year post birth, is not only reassuring, but also produces a significant insight that neither study alone had addressed: a comparison of MC to WB responses. For cord blood vs. adult peripheral blood we had previously completed the direct MC vs. WB comparison, and found there to be striking and significant qualitative and quantitative differences in TLR responses between neonatal vs. adult MC and WB [10]. The fact that our current observations using MC and those of Nguyen *et al.* who used WB [18] uncovered the same trends over the first year of life suggests that the impact of soluble factors, such as adenosine [8], [25] or other cells contained in WB on the TLR response, while important around birth [8], may be less important by 1-year of age. Another study investigating the innate responses to TLR stimulation from newborn up to 1-mo old infants confirmed, in a direct comparison of MC to WB, that the impact of soluble factors or other cells contained in WB (as compared to MC) on TLR responses disappears by a month after birth [19]. However, *in vivo* exposure to e.g. different concentrations of adenosine may still influence our *in vitro* results, as may age-dependent differences in the sensitivity of infant mononuclear cells to adenosine [25].

Belderbos *et al.*, similar to our and Nguyen's observations, also describes a rapid postnatal increase in IFN-α2 production in response to TLR7/8 stimulation, and an LPS-induced increase in IL-12p70 production by 1 month but only to levels still below those of the adult [19]. However, they analyzed the cytokine response in WB culture supernatant after stimulation with LPS following IFN-γ priming, and used concentrations of pI:C and CpG-A much higher than ours. The latter aspect, the need for higher pI:C and CpG-A concentrations when stimulating WB vs. MC is consistent with our previously made observation of a lack of response of APC in WB at concentrations that maximally stimulate APC in MC [9], [10]. Using this much higher concentration of pI:C in WB cultures, Belderbos *et al.* describe a rapid increase to adult levels of IL-12p70 production by 1-mo of age [19]. While we also detected an increase in pI:C-induced IL-12p70 production postnatally, we did not see this level reach those of adults even by 2- years of age. This difference may relate to the higher concentration of pI:C, or may indicate changes between 1-mo and 1-2-years of life. The observation by Belderbos *et al.* regarding TLR9 induced IL-10 production is in line with our and Nguyen's observation, in that it appears to rapidly drop off from a neonatal high [18], [19]. Similar to Belderbos *et al.*, we did not detect a drop in IL-10 production in response to LPS stimulation from the high cord levels by 1 year of age; we did however observe a striking drop of IL-10 produced in response to LPS to below adult levels between 1- and 2-years of age.

Two other studies investigating the postnatal ontogeny of innate immune responses in humans come from the same group [14], [20]. Similar to us, this group used MC; but their MC were cryopreserved and thawed prior to TLR stimulation, while ours were stimulated fresh within 4 hours of collecting the blood. Furthermore, this group stimulated with LPS only after IFN-γ priming, while our stimulations occurred without prior priming. Despite these differences in experimental setup, Upham *et al.* describe an IL-12p70 production pattern consistent with our findings, in that even by 12 years of age, IL-12p70 appears to still not be produced at levels comparable to adults, while early life elevated IL-10 production reaches the low adult level by at least 5 years of age [20]. Yerkovich *et al.* observe a level of IL-6 and IL-10 production at birth that is similar to the adult, but drops off by 1- and even further by 2-years of age [14], which agrees with our findings. Neither Yerkovich *et al.* nor Upham *et al.* used any of our other TLR stimuli, limiting the extent to which these studies can be compared to ours [14], [20].

Yerkovich *et al.* and Upham *et al.* are also the only other studies that attempt to identify the cellular source of the cytokines produced in postnatal samples after TLR stimulation. Upham *et al.* approached this through depletion, and determined that most of the IL-12p70 after LPS/IFN-γ priming is made by dendritic cells [20]. Yerkovich *et al.* employed ICS, but limited staining to only 4 parameters, namely CD3, CD14, TNF-α and IL-6. Similar to our ICS results, they determined that monocytes in cord blood produce high levels of both, TNF-α and IL-6 [14]. We are not aware of any other study that conducted the extensive polychromatic single-cell TLR induced cytokine analysis via intracellular cytometry that we describe here; our approach to combine this type of ICS with the bulk culture supernatant analysis is thus the most comprehensive innate immune profiling study in early life to date. Interestingly, our findings comparing ICS and multiplex bead-array were largely congruent. For example, neonatal monocytes in MC samples contained higher percentages of cells making IL-12/23p40 than adult monocytes, but by 1 year of age, this had started to drop for both, ICS and for secreted IL-12/23p40 measured via multiplex bead-array. The same was true for IL-6 production. Similarly, monocytes and cDCs from 1- and 2- year olds contained higher percentages of cells making TNF-α after TLR2/1 or TLR4 stimulation as compared to the neonatal sample of the same subjects; this was also the case analyzing the culture supernatant for TNF-α. And lastly, the amount of IFN-α2 detected in the culture supernatant after TLR7/8 or TLR9 stimulation increased dramatically from birth to reach or exceed adult levels by 1-year of age; as IFN-α2 is mostly produced by pDC, this increase in IFN-α2 production was also detected by ICS in pDC. We thus were able to confirm, at the single-cell level our observations made in the bulk-culture supernatants.

Our *in vitro* findings with innate cells from newborns and infants found in blood and after exposure to adult serum may not accurately reflect the response to microbes or vaccine adjuvants found in tissues of an infant. However, several observations suggest that our findings described in this study are at least consistent with clinical presentations of the human newborn and infant. For example, infection with HSV results in the most severe morbidity and mortality if it occurs before 1-month of life [26]; as it is IFN-α2 that is most relevant to protection from HSV infection, the rapid increase in IFN-α2 production early in life may explain this clinical observation [26]. The delay in IFN-γ and IL-12p70 production beyond 2 years of life is consistent with the known heightened susceptibility beyond 2 years of age to infection with microbes such as TB, where protection is known to depend on robust IFN-γ and IL-12p70 production [13]. The change of the age-specific predominant innate immune response we observed may also impact vaccine immune response preferences. For example, BCG immunization at birth has been shown to support a stronger Th17 adaptive T cell response as compared to BCG given at 4 months of age [27], while a delay in BCG vaccination for several months post-birth results in a somewhat stronger Th1 type T cell response [28]; this observation is entirely consistent with the preferential production of Th17 supporting innate cytokines around birth we detected.

In summary, we present here the results of the most comprehensive longitudinal innate immune profiling study covering the first 2 years of human life. Our findings contradict the notion that the ontogeny of the innate immune response to TLR stimulation linearly progresses from birth to adulthood. Instead, it appears that age-specific regulatory mechanisms are in place governing the TLR response by the major human APC subsets. With the precise knowledge of the most pertinent ontogenic changes in TLR responses in hand, we are now well positioned to initiate a targeted interrogation of the underlying molecular mechanisms governing early life innate immune ontogeny. This, in turn, will form the rational basis on which to attempt age-specific and age-appropriate interventions aimed at improving the immune-mediated protection of this highly vulnerable population.

## Materials and Methods

### TLR stimulation

TLR stimulation plates were prepared as described previously [9]. Briefly, deep-96-well (VWR) source plates containing 1.3 ml of various TLR ligands at 10 times the desired concentration were prepared using sterile procedures under a laminar airflow hood. The following TLR ligands were used at the concentrations noted in the figure or table legends: PAM_3_CSK_4_ (PAM; TLR2/1; EMC Microcollections); poly(I:C) (pI:C; TLR3; Amersham Biosciences); 0111:B4 LPS (LPS; TLR4, InvivoGen); 3M-003 (3M-003; TLR7/8, 3M); ODN 2336 (CpG-A; TLR9; Coley). For the 6-h ICS plates, brefeldin A (BFA; Sigma-Aldrich) was added at a concentration of 100 µg/ml (10 times the desired final concentration of 10 µg/ml) to all wells, except those wells containing TLR3 or 9 ligands. BFA was not added to the 10× source plates for the plates that were used to obtain 18-h supernatants for Luminex and ELISA cytokine quantification. Source plates were sealed with sterile aluminum plate sealers (USA Scientific), frozen at -80°C, and thawed before use. Twenty microliters from each well of the source plate was dispensed into each well of recipient 96-well round-bottom polystyrene plates (Corning) using the Evolution P3 Precision Pipetting Platform (PerkinElmer) under a laminar airflow hood using sterile procedures. Recipient plates were sealed with sterile aluminum plate sealers and frozen at −80°C until use.

### Birth cohort study design

All studies were approved by the Institutional Ethics Review Board at both the University of Washington and the University of British Columbia. Our longitudinal study was set-up specifically for the purpose of analyzing the ontogeny of the human innate immune system over the first 2 years of life. Parents were contacted prior to birth at the BC Children's hospital, and written informed consent was obtained from both parents. Only healthy, full-term newborns after elective Caesarean sections without labor were enrolled in this study between 2005 and 2008. Prior to each postnatal blood draw, written informed consent was obtained again from the parent or legal guardian. The infant's health was assessed via a history-driven health assessment (HDHA), focusing on past or current chronic or acute abnormalities or medical conditions. Infants were excluded from the entire study if any the following exclusion criteria were met at any of time of the study: (1) The diagnosis of a significant chronic medical condition including: HIV infection; immune deficiency; immunosuppression by disease or medication; cancer; bone marrow or organ transplantation; blood product administration within the last 3 mo; bleeding disorder; known congenital malformation or genetic disorder; (2) If the parent or legal guardian were unable to read and/or comprehend English; (3) if the family moved outside of the Greater Vancouver Area during the study period (i.e., would be unavailable for follow-up). Additionally, any febrile illness within the last 24 h, recent immunizations (within 4 weeks of live vaccine, or 7 days after non-live vaccines), or brief (<1 mo) immunosuppressive medication use within one month, would result in a deferral of the blood draw to a later date. Subjects in the birth cohort were 55% female and 45% male; 54% percent of our birth cohort subjects were identified by their parents as having Caucasian ancestry; 32% as having Asian ancestry; 8% as having African American ancestry; 2% as having Native American/First Peoples ancestry; 2% as having Native Hawaiian/Pacific Islander ancestry; 2% as having Hispanic ancestry. This distribution is representative of the American Northwest/Canadian Southwest coasts from which it was randomly sampled. Healthy adults, unrelated to the infants, aged 23 to 48, of equal male-female ratio were recruited from the same area, and had a similar ethnic background as our infant study subjects. All blood draws were performed in the hospital by a trained pediatric phlebotomist. Peripheral blood (7–10 ml) was drawn via sterile venipuncture into Vacutainers containing 143 USP units of sodium-heparin (Becton Dickinson (BD) Biosciences, catalog no. 8019839) using batches we had previously confirmed to be free of innate immune activating substance in assays performed as described elsewhere [9].

### Blood sample processing and in vitro stimulation

Blood samples were processed as described previously [9]. Neonatal cord blood or adult peripheral blood mononuclear cells (MC) were isolated by Ficoll-Paque density gradient centrifugation. MC were cultured in RPMI 1640 supplemented with 100 U penicillin/ml, 100 mg streptomycin/ml (Invitrogen), and 10% human AB serum (Gemini Bio-Products). Two hundred microliters of cell suspension (2.5×10^6^ MC/ml) was added to each well of the premade plates containing the specific TLR ligands. For the ICS assays, cells were incubated for 6 h at 37°C in 5% CO_2_. For the TLR3 and TLR9 ligands, BFA was added 3 h later at a final concentration of 10 µg/ml, which provides optimal detection of intracellular cytokine production in response to these ligands [9]. After culture, cells were treated with a final concentration of 2 mM EDTA for 15 min at 37°C, then spun down and resuspended in 100 µl of 1x BD FACS Lysing Solution, sealed, and stored frozen at −80°C until staining. An identical set of plates was incubated in parallel for 18 h without BFA; at 18 h, these plates were spun and 100 µl of supernatant was removed and frozen at −80°C for later Luminex analysis.

### Staining, acquisition, and analysis

Preparation of the samples for flow cytometric analysis was performed as described previously [9], [10], [11]. A detailed description of Ab (source, clone, and dilution), machine set up, and data acquisition compliant with the recently accepted MiFlowCyt reporting standards [11], [29] can be found in the Text S1. Briefly, frozen plates were thawed and spun, and pellets were resuspended in 200 µl of BD FACS Permeabilizing Solution and incubated at room temperature for 10 min. After one wash in PBS containing 0.5% BSA and 0.1% sodium azide (PBSAN), cells were stained in a final volume 100 µl of PBSAN for 30–60 min at room temperature. After two additional washes with PBSAN, cells were resuspended in PBS containing 1% paraformaldehyde and immediately analyzed on an LSRII Flow Cytometer (BD Biosciences) set up according to published guidelines [9], [11], [30]. Compensation beads (CompBeads; BD Biosciences) were used to standardize voltage settings and used as single-stain positive and negative controls as described previously [9], [11], [31]. A total of 200,000 events were acquired for adult and neonatal MC. Compensation was set in FlowJo (Tree Star) and samples were analyzed compensated. Gates were set based on the fluorescence-minus-one principle [15], [32]. We positioned the unstimulated flow cytometric samples as a biological negative control; this has been identified as the most appropriate approach for flow cytometric analysis of stimulation experiments [15].

### Assessment of cytokines in culture supernatant

Supernatants were thawed at room temperature, and then filtered through a 1.2-µm filter plate (Millipore) into a clean 96-well plate to remove any remaining cellular debris using a multi-screen HTS vacuum manifold (Millipore). The Luminex assay was performed using the Upstate/Millipore “Flex Kit” system using the high-biotin protocol and overnight incubation at 4°C. Cytokines measured were IL-6, IL-10, IL-12p40, IL-12p70, TNF-α, IFN-α, and IFN-γ. Samples were diluted 1-to-1 (and, if needed to fall within the standard curve, 10- or 20-fold) with RPMI 1640 supplemented 10% human AB serum. Beadlytes, biotin, and streptavidin-phycoerythrin, were used at half the manufacturer's recommended concentrations. Assays were read using Luminex 200 Total System (Luminex) running either the Bio-plex (Bio-Rad) or the MasterPlex (MiraiBio) softwares, and the downstream analysis was performed using Excel (Microsoft) and an in-house database. To determine the IL-23 concentration, filtered supernatants were diluted 1∶4 in diluent contained in the human IL-23 (p19/p40) ELISA kit (eBioscience), and assays were performed according to the manufacturer's specifications. Plates were read at 450 nm with 570-nm subtraction. A sigmoid logistic curve was used to generate the standard curve. To allow assessment of the level of cytokine production detected in culture supernatants of unstimulated samples data was not subtracted from the stimulated samples but shown side by side, as this data is possibly biologically relevant [33], [34], [35].

### Statistical analysis

raphs were prepared using Excel (Microsoft). Statistical differences between neonate-infant pairs were analyzed using the Wilcoxon matched-pair signed rank test, while the Mann-Whitney unpaired test was used to compare the neonates/infants to the adults. To correct for multiple comparisons, we computed the Bonferroni corrected acceptable Type I error rate (alpha) as 0.05/6 = 0.01 (0.0083), as these would yield false discoveries at a rate of <0.05 comparing cord blood to 1- and 2- year old infant samples as well as adult, 1 year infant samples to 2 year old infant and adult samples, and 2 year old infant samples to adult (i.e. a total of 6 comparisons for each data set) [36]. Accordingly, we consider p-values of less than 0.01 to indicate a significant difference. To assess polyfunctionality (i.e., the ability of an individual cell to produce one vs. more than one cytokine in response to a specific stimulus), we computed the percentage of cells in a given cytokine-combination category (e.g., there are 7 possible cytokine-combination categories for 3 cytokines in which at least one cytokine is positive (2^3^−1 = 7)). The percentages of reactive cells (i.e., cytokine-producing cells) that were positive for only one of the 3 cytokines were added up to give the polyfunctional degree (PFD) 1 (PFD1); and separately, the percentages of reactive cells expressing any 2 of the 3 cytokines or all 3 cytokines were computed to give PFD2 and PFD3 values, respectively. Given that PFD values represent qualitative composites of several categories of quantitative data, and statistical analysis would thus be of little value, we did not subject the PFD analysis to statistical examination.

## Supporting Information

Figure S1 **Gating strategy to identify antigen-presenting cell subsets in cord, 1- year, 2-year and adult blood sample.** Gates for monocytes (MHC-II^+^, CD14^+/high^), conventional DC (MHC-II^+^, CD14^−/low^ then CD123^−^, CD11c^+^), plasmacytoid DC (MHC-II^+^, CD14^−/low^ then CD11c^−^, CD123^+^) and B cells (MHC-II^+^, CD14^−/low^ then CD11c^−^, CD123^−^) are shown.
(TIF)

Figure S2 **Percentage composition of total cells acquired in flow cytometric analysis for monocytes, B cells, cDC, and pDC.** Relative percentage of MC cell populations in cord blood, and peripheral blood at 1 and 2 years of age, in comparison to adult controls. The mean for each age group is indicated by the middle line, with error bars indicating standard deviation. Statistically significant (p<0.01) differences between age groups are indicated by *.
(TIF)

Figure S3 **An example of intracelluar cytokine cytometry analysis illustrating the extent of change in cytokine expression after TLR stimulation in the indicated APC cell subsets from cord, 1-year, 2-year, and adult mononuclear cell.** An overlay is used to compare the unstimulated sample (blue) with the sample stimulated (red) with the TLR7/8 ligand, 3M-003, for the same subject in each age group.
(TIF)

Table S1 **Flow cytometric comparison of percent of cells expressing cytokines for longitudinal neonatal and adult responses to TLR stimulation.** Statistical analysis of the average percentage of a given gated cell population (monocytes, cDC, or pDC) that express a given cytokine in intracellular flow cytometric analysis. Statistically significant (p<0.01) differences between age groups are indicated in bold type-set. Mean percent positive cells for each population are derived from the FloJo software, with variance represented as standard deviation calculated using Prism 5. ND  =  not detectable.
(XLSX)

Table S2 **Flow cytometric comparison of the mean fluorescent intensity for longitudinal neonatal and adult responses to TLR stimulation.** Statistical analysis of average mean fluorescent intensity (MFI) of the given gated cell population (monocyte, cDC, or pDC that express a given cytokine in intracellular flow cytometric analysis). Statistically significant (p<0.01) differences between age groups are indicated in bold type-set. MFIs for each population are derived from the FloJo software, with variance represented as standard deviation calculated using Prism 5. ND  =  not detectable.
(XLSX)

Table S3 **Comparison of the concentrations of secreted cytokines for longitudinal neonatal and adult responses to TLR stimulation.** Cytokines secreted MC in tissue culture media containing the indicated TLR ligands were determined in pg/ml for 35 neonate-infant subjects and 25 adult subjects. Statistically significant (p<0.01) differences between age groups are indicated in bold type-set. NP  =  not performed.
(XLSX)

Text S1 **MIFlowCyt standard compliant information for submitted flow cytometric data.** A detailed description of the antibodies (source, clone, and dilution), machine set up, and data acquisition for the flow cytometry experiments described in this paper.
(DOC)
