# Supplementary figures and images for: Ontogeny of Toll-Like Receptor Mediated Cytokine Responses of Human Blood Mononuclear Cells
Source: PLoS One. 2010 Nov 30;5(11):e15041. doi: 10.1371/journal.pone.0015041 (PMC2994830; doi:10.1371/journal.pone.0015041)

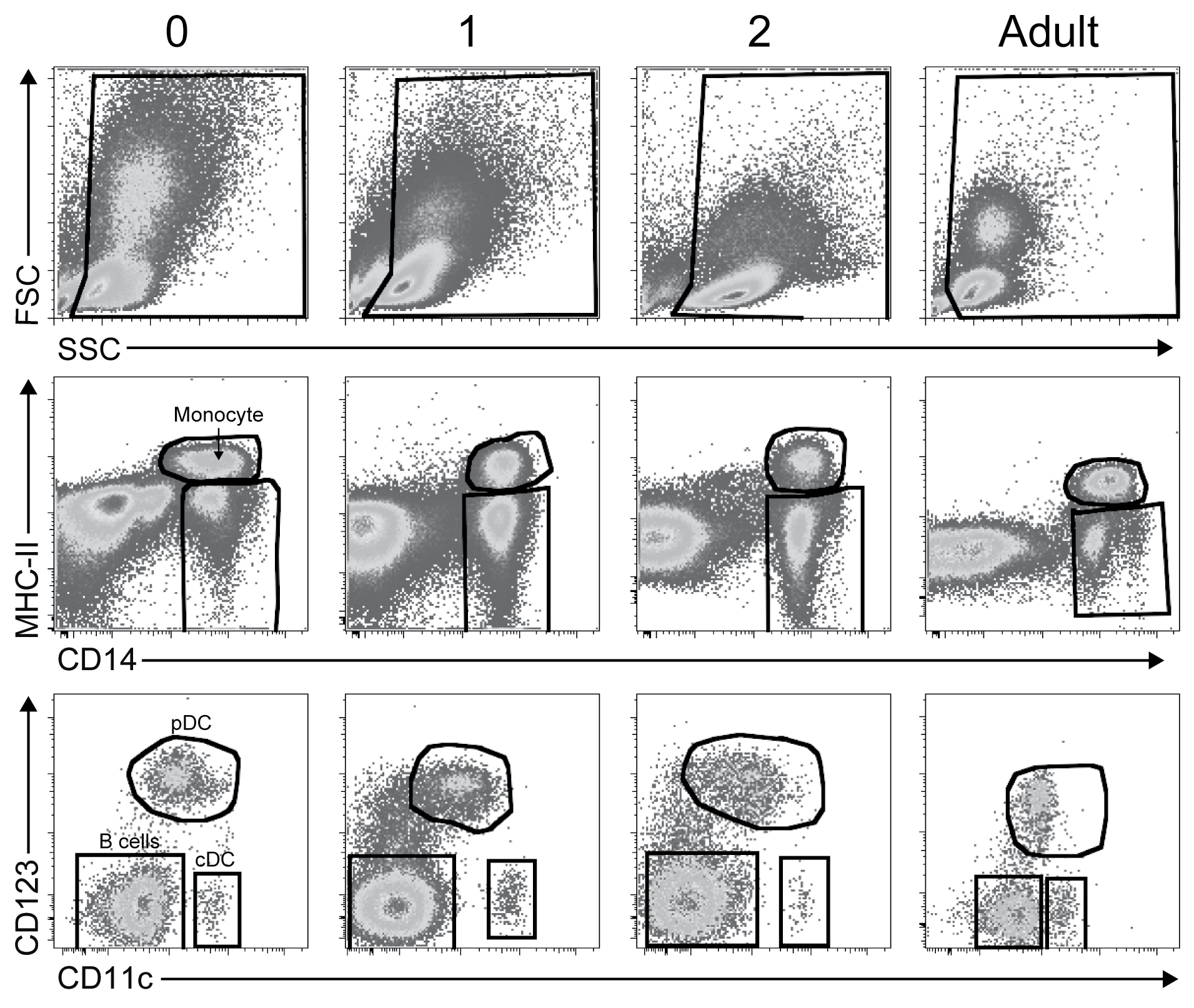

Supplement: Figure S1 — Gating strategy to identify antigen-presenting cell subsets in cord, 1- year, 2-year and adult blood sample. Gates for monocytes (MHC-II+, CD14+/high), conventional DC (MHC-II+, CD14−/low then CD123−, CD11c+), plasmacytoid DC (MHC-II+, CD14−/low then CD11c−, CD123+) and B cells (MHC-II+, CD14−/low then CD11c−, CD123−) are shown. (TIF) [file pone.0015041.s001.tif]

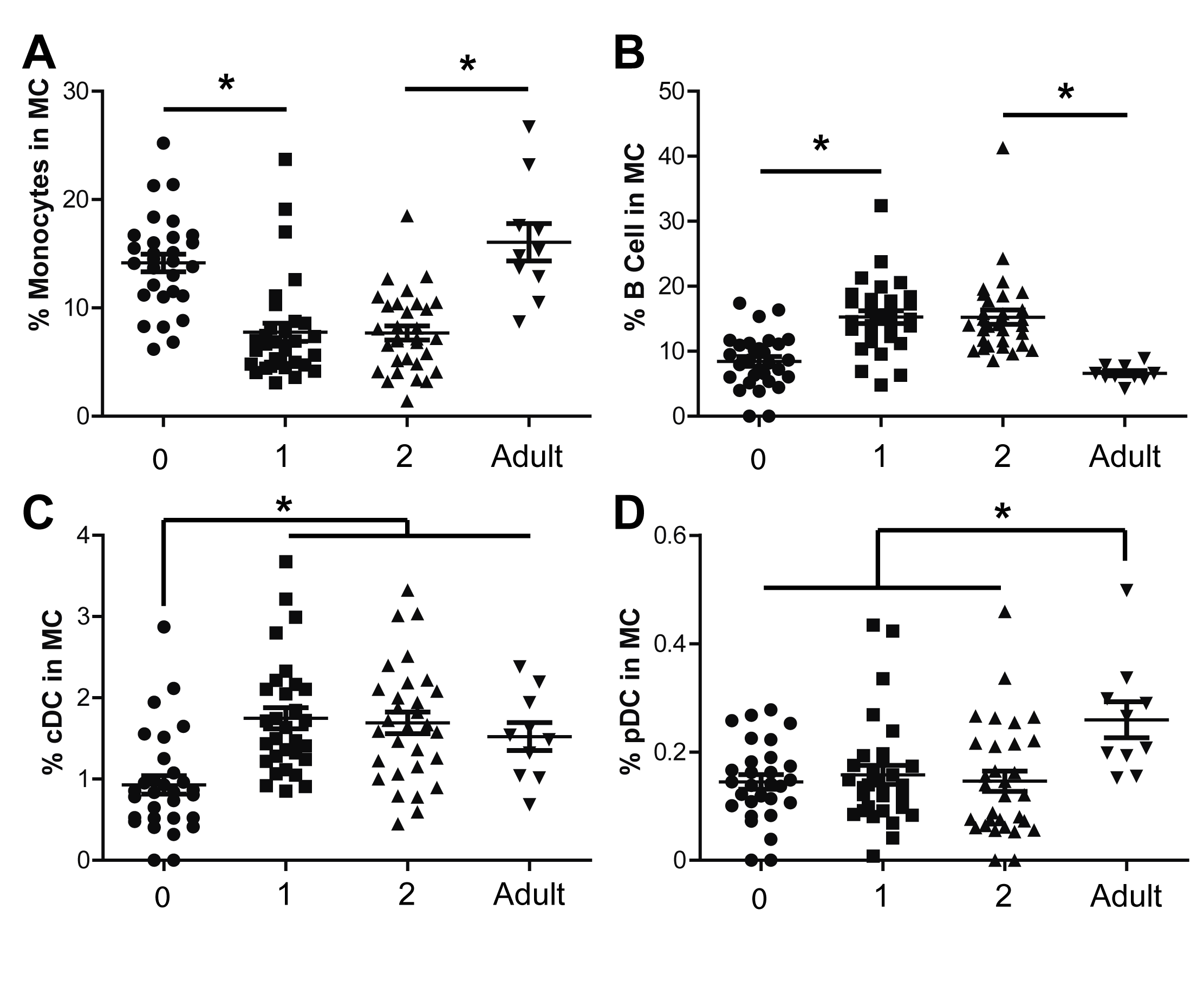

Supplement: Figure S2 — Percentage composition of total cells acquired in flow cytometric analysis for monocytes, B cells, cDC, and pDC. Relative percentage of MC cell populations in cord blood, and peripheral blood at 1 and 2 years of age, in comparison to adult controls. The mean for each age group is indicated by the middle line, with error bars indicating standard deviation. Statistically significant (p<0.01) differences between age groups are indicated by *. (TIF) [file pone.0015041.s002.tif]

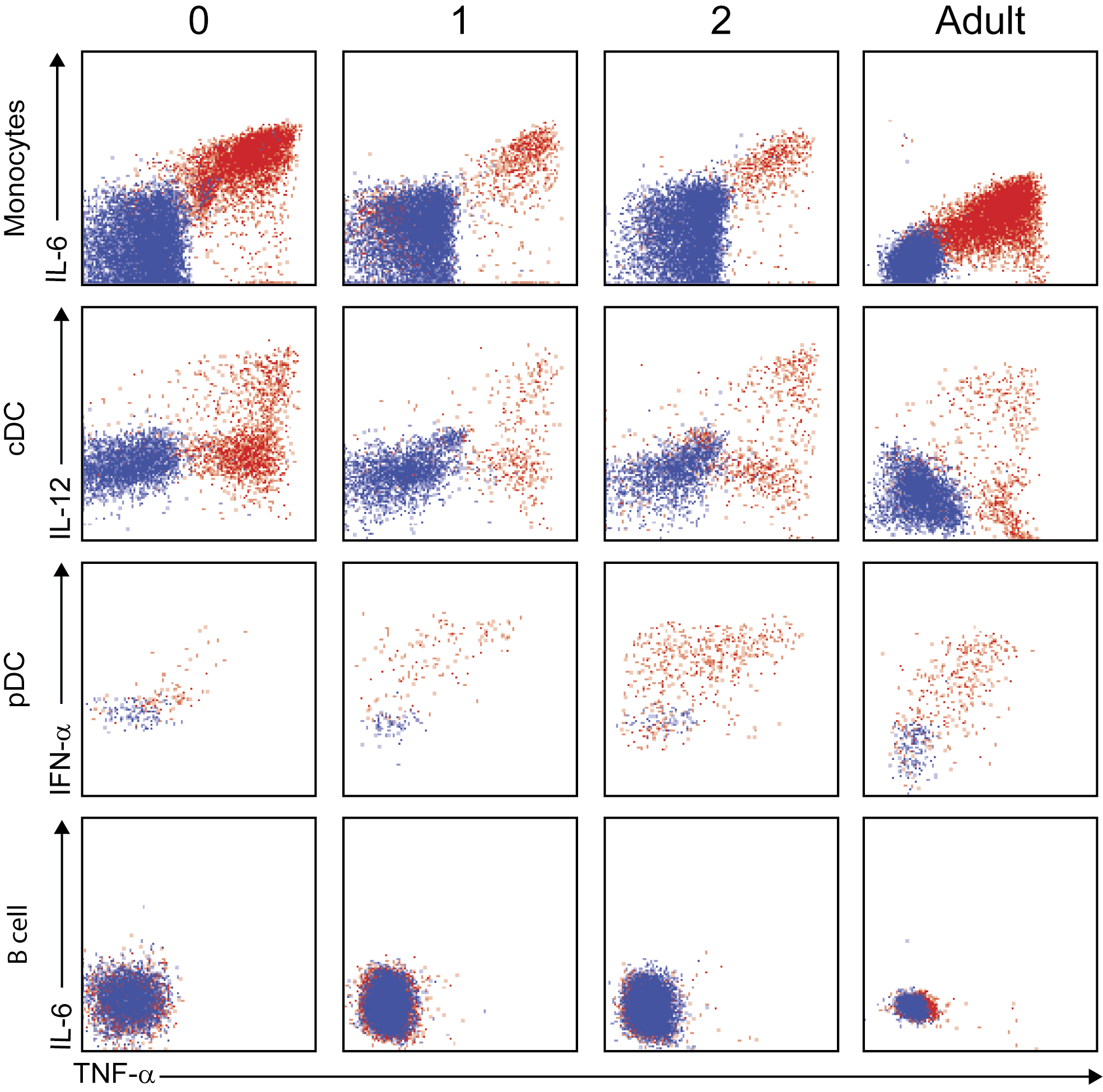

Supplement: Figure S3 — An example of intracelluar cytokine cytometry analysis illustrating the extent of change in cytokine expression after TLR stimulation in the indicated APC cell subsets from cord, 1-year, 2-year, and adult mononuclear cell. An overlay is used to compare the unstimulated sample (blue) with the sample stimulated (red) with the TLR7/8 ligand, 3M-003, for the same subject in each age group. (TIF) [file pone.0015041.s003.tif]
