# Supplementary material for: Ontogeny of Toll-Like Receptor Mediated Cytokine Responses of Human Blood Mononuclear Cells
Source: PLoS One. 2010 Nov 30;5(11):e15041. doi: 10.1371/journal.pone.0015041 (PMC2994830; doi:10.1371/journal.pone.0015041)
Supplement: Text S1 — MIFlowCyt standard compliant information for submitted flow cytometric data. A detailed description of the antibodies (source, clone, and dilution), machine set up, and data acquisition for the flow cytometry experiments described in this paper. (DOC) [file pone.0015041.s007.doc]

**Supplemental Data**

Corbett *et al.*, Ontogeny of the Innate Immune Response to Toll-like Receptor Stimulation

MIFlowCyt standard compliant information for submitted flow cytometric data.

**1. Experiment overview.**

**1.1. Purpose**: The purpose of this experiment was to track the changing responses of monocytes, B cells (by negative-gating), plasmacytoid dendritic cells, and myeloid dendritic cells to TLR stimulation during the first two years of life. Comparison to adult peripheral blood was performed to give an idea as to the relationship between adult and changing early life TLR responses. As previous work had accomplished comparison between whole blood and PBMC fractionated cell populations, only PBMCs were the focus of this study. We hypothesized that–given the changes in environment and ontogeny–PBMC TLR responses would vary over time in the very young, and that this knowledge could form the basis of future work in rational vaccine design for the pediatric field.

**1.2. Keywords:**

**1.3. Organization:**

**1.3.1.** Kollmann Lab, University of British Columbia

**1.3.2.** 938 W28th Ave. Vancouver, British Columbia, V5Z 4H4, Room: A5-151

**1.4. Primary Contact:**

**1.4.1.** P.I. Dr. Tobias Kollmann [tkollmann@cw.bc.ca](mailto:tkollmann@cw.bc.ca)

**1.4.2.** Research Associate. Dr. Edgardo Fortuno III [fortuno@interchange.ubc.ca](mailto:fortuno@interchange.ubc.ca)

**1.4.3.** Graduate Student. Mr. Nathan Corbett [ncorbett@cfri.ca](mailto:ncorbett@cfri.ca)

**1.4.4.** Research Assistants: Darren Blimkie [dblimkie@interchange.ubc.ca](mailto:dblimkie@interchange.ubc.ca); Juliet Crabtee jnc3@u.washington.edu; Annie Rein-Weston arein@u.washington.edu.

**1.5. Date:** Experiments were **set up from 5/24/07 to 5/25/10 and stained from 6/6/07; to 6/25/10.**

**1.6. Conclusions:**

Responses to TLR ligands change significantly from birth to 2 years of age. For some indicators of TLR response and certain TLR stimuli (e.g. IFN-α2 in TLR8/9 stimulation, TNF-α in TLR4 stimulation), levels approached those observed in similarly treated adult cells by 1 year of age. Conversely, others made an apparent decline from birth to 2 years of age (e.g., IL-6), indicating an alternate TLR response by the very young. It is not yet known how or when these responses reach adult-like status. In sum, the neonatal immune system is distinct from that of the adult, and develops through age-specific phases instead of linear trend towards an adult-like pattern.

**1.7. Quality Control Measures:**

Unstimulated controls were set up for each condition tested. Single stain controls were set up by staining 3 ul of Anti-Mouse Ig CompBeads (BD #552843) and 3 ul of anti-FBS negative control beads (included with BD #552843) with 3ul of each antibody used.

**2. Flow Sample/Specimen Description**

**2.1. Sample/Specimen Material**

**2.1.1. Biological Samples:**

**2.1.1.1. Biological Sample Name**

Cord and adult mononuclear cells

**2.1.1.2.Biological Sample Source:** Healthy human adult peripheral blood; cord blood obtained and processed within < 4h from a healthy full-term baby born via C-section (no labor); healthy infant peripheral blood obtained and processed within < 4h.

**2.1.1.2.1. Biological Sample Source Organism:**

**2.1.1.2.1.1. Taxonomy:**

Homo Subspecies sapiens

**2.1.1.2.1.2. Age:**

Birth (for cord blood) to 2 years of age (venous blood); 21-45 years old (adult venous blood).

**2.1.1.2.1.3. Gender:**

Male and Female.

**2.1.1.2.1.4. Phenotype:**

Healthy (none).

**2.1.1.2.1.5. Genotype:**

Not Applicable.

**2.1.1.2.1.6. Treatment:**

PBMCs were isolated from peripheral blood using Ficoll gradient centrifugation. After purification, cells were washed twice with DPBS then resuspended at 2.5E6 cells/ml in RPMI supplemented with 10% human AB serum and 1% Penicillin/Streptomycin.

**2.1.2. Environmental Samples:** Not Applicable.

**2.1.3. Control Sample Description:**

Single stain controls were set up by staining 3 ul of Anti-Mouse Ig CompBeads (BD #552843) and 3 ul of anti-FBS negative control beads (included with BD #552843) with 3ul of each antibody used. Also, for cytokine analysis, fluorescence minus one (FMO) staining was applied, in which the antibody for the cytokine of interest is omitted from the normal antibody staining panel.

Lastly, unstimulated control were used as controls to set analysis gates.

**2.1.4 Sample Treatment Description**

Cells were plated in a 96 well plate and cultured for a total of 6 hrs. Cells were stimulated with either nothing, or PAM3CSK4 (TLR2/1, EMC Microcollections); poly I:C (TLR3, Amersham); 0111:B4 LPS (TLR4, Invivogen); 3M-003 (TLR7/8, 3M); CpGA (TLR9, Coley). After culture, cells were treated with a final concentration of 2mM EDTA for 15 min at 37°C, then centrifuged @400g for 5min @22˚C and resuspended in 100 ul of 1x BD FACS Lysing solution (BD 349202) for 10 minutes at room temperature before being frozen at -80°C.

**3. Fluorescence Reagent Description:**

Table 1.

|  | **Characteristic Being Measured** | **Antibody Name *Clone Name*** | **Vendor cat# *dilution used*** |
| --- | --- | --- | --- |
| **VIOLET** |  |  |  |
| **AmCyan/ PO** | Cell Surface Protein | CD123 AmCyan (9F5) | BD#custom *1:50* |
| **Pacific Blue** | Intracellular Protein | IL12p40/70 *(eBio: C8.6)* | eBio#577129 *1:100* |
| **RED** |  |  |  |
| **APC** | Cell Surface Protein | CD11c (5HCL3) | BD#340714 *1:50* |
| **APC-Cy7** | Intracellular Protein | IL6 *(AS12)* | BD #custom *1:100* |
| **Alexa 700** | Intracellular Protein | TNFa *(Mab11)* | BD#557996 *1:100* |
| **BLUE** |  |  |  |
| **FITC/OG** | Intracellular Protein | IFNa *(A11)* | Antigenix#MC100133 *1:100* |
| **PerCPCy5.5** | Cell Surface Protein | MHCII *TU36* | BD#custom *1:100* |
| **PE-Cy7** | Cell Surface Protein | CD14 (M5E2) | BD #557742 *1:50* |

**Instrument Details:**

**3.1. Manufacturer:**

**BD Biosciences**

**3.2. Model:**

BD LSR II 4 Laser, Blue/Red/Violet/UV cat # 347545

**3.3. Instrument Configuration and Settings:**

All lasers, filters and mirrors were manufactured by BD Biosciences. All filters

and mirrors came with the machine and were installed March 2005.

**3.3.1. Light Sources:**

The light path, filters and detectors are described below in Table 2. The lasers are listed in the order the cells pass through them. The detectors and filters are listed in the order the light hits them, with the exception of FSC which is measured from light that passes through the cell/bead while all the other 488 nm detectors detect light that has been scattered 90°, in the order listed. For example, for blue laser detector A light passes through or is reflected off of filter 1, 735 LP, then the light passes through filter 2, 780/60 BP, then it hits the PMT detector. Light that is reflected off the long pass goes to detector B and so on. For parameters used in this experiment, it is indicated whether Area (-A), Height (-H) or Width (-W) was used.

Abbreviations:

PMT = photomultiplier tube

PD = photodiode,

BP = band pass filter, first number is center of interval, second number is the

width of the interval.

LP = long pass filter, lets light waves through that have a longer wavelength

than the number specified. All LP filters are dichroic and reflect at an angle

of incidence at 11.25°.

Table 2.

| **Laser** | **Detector Name (Type)** | **Filter 1 (1st filter)** | **Filter 2 (2nd Filter)** | **Parameter detected** | **Detector voltage** | **Amplification Type** |
| --- | --- | --- | --- | --- | --- | --- |
| **Blue Laser (488 nm)** | FSC (PD) | 488/10 BP | na | FSC-A | 390 | LINEAR |
| Solid state Coherent Sapphire blue laser 20 mW | 488 A (PMT) | 735 LP | 780/60 BP | PE-Cy7-A | 625 | LOG |
|  | 488 B (PMT) | 685 LP | 712/21 BP | PerCP-Cy5.5-A | 750 | LOG |
|  | 488 C (PMT) | 655 LP | 670/14 BP | PerCP | na |  |
|  | 488 D (PMT) | 595 LP | 610/20 BP | PE-TexRed | na |  |
|  | 488 E (PMT) | 550 LP | 575/26 BP | PE | na |  |
|  | 488 F (PMT) | 505 LP | 530/30 BP | FITC, Ax488-A | 450 | LOG |
|  | 488 G (PD) | blank | 488/10 BP | SSC-A | 410 | LINEAR |
|  | 488 H (PMT) | blank | na | blank | na |  |
| **Violet Laser (405 nm)** | 405 A (PMT) | 505 LP | 585/42 BP | AmCyan-A | 650 | LOG |
| Coherent VioFlame PLUS laser 25mw | 405 B (PMT) | blank | 440/40 BP | Pacific Blue-A | 530 | LOG |
|  | 405 C (PMT) | blank | na | blank | na |  |
| **UV Laser (355 nm)** | 355 A (PMT) | 505 LP | 530/30 BP | Ca++ Blue | na |  |
| Solid state Coherent Lightwave Xcyte 20mW | 355 B (PMT) | blank | 440/40 BP | Alexa350 | na |  |
|  | 355 C (PMT) | blank | na | blank | na |  |
| **Red Laser (637 nm)** | 637 A (PMT) | 755 LP | 780/60 BP | APC-Cy7-A | 700 | LOG |
| Solid State Coherent laser 25 mW | 637 B (PMT) | 685 LP | 710/50 BP | Alexa700-A | 600 | LOG |
|  | 637 C (PMT) | 660/20 BP | na | APC-A | 505 | LOG |

**4. Data Analysis**

**4.1. FCS Data File:**

To request raw data please contact Dr. Tobias Kollmann [tkollmann@cw.bc.ca](mailto:tkollmann@cw.bc.ca)

**4.1.1. Total Count of Events:**

Recorded within individual FCS files, 200 000 events per tube.

**4.2. Compensation Description:**

Compensation was done in FlowJo using BDCompBeads as single stain controls.

The matrix is below in Table 3:

Compensation Matrix for one of the sample shown in Supplementary Figure1.

**4.3. Gating (Data Filtering) Description:**

**4.3.1. Gate Summary Information:**

**4.3.1.1. -4.3.1.3 Gate Descriptions/subpopulations/statistics:**

|  |  | **Gate Statistics (% Parent Gate)** | |
| --- | --- | --- | --- |
| **Gate Description:** | **Qualitative Description of the Subpopulation** | **Unstim** | **R848 stim** |
| Live Cells | High cell density excluding lower left corner population | 79.6 | 71.9 |
| Monocytes | CD14 high, MCHII high | 24.9 | 13.2 |
| Other MHCII+ cells | MHCII high, CD14 mid to low | 15.3 | 19.2 |
| Myeloid Dendritic Cells (cDCs) | MHCII high, CD11c high, CD123 low | 22.9 | 26.9 |
| Plasmacytoid Dendritic Cells (pDCs) | MHCII high, CD11c low, CD123 high | 2.39 | 2.42 |
| Monocyte TNF+ IL-6 ­ | "Monocyte" TNFa high, IL-6 low | 0.29 | 16.6 |
| Monocyte TNF+ IL-6+ | "Monocyte" TNFa high, IL-6 high | 0.15 | 46.7 |
| Monocyte TNF- IL-6+ | "Monocyte" TNFa low, IL-6 high | 0.28 | 16.6 |
| Monocyte TNF- IL-6­ | "Monocyte" TNFa low, IL-6 low | 99.3 | 20.1 |
| Monocyte TNF+ IL-12­ | "Monocyte" TNFa high, IL-12 low | 0.35 | 61.8 |
| Monocyte TNF+ IL-12+ | "Monocyte" TNFa high, IL-12 high | 0.093 | 1.52 |
| Monocyte TNF- IL-12+ | "Monocyte" TNFa low, IL-12 high | 0.048 | 0.15 |
| Monocyte TNF- IL-12­ | "Monocyte" TNFa low, IL-12 low | 99.5 | 36.5 |
| Monocyte TNF+ IFNa ­ | "Monocyte" TNFa high, IFNa low | 0.43 | 63.2 |
| Monocyte TNF+ IFNa+ | "Monocyte" TNFa high, IFNa high | 0.00757 | 0.074 |
| Monocyte TNF- IFNa+ | "Monocyte" TNFa low, IFNa high | 0.033 | 0.079 |
| Monocyte TNF- IFNa­ | "Monocyte" TNFa low, IFNa low | 99.5 | 36.6 |
| cDC TNF+ IL-6 ­ | "cDC" TNFa high, IL-6 low | 0.98 | 32.2 |
| cDC TNF+ IL-6+ | "cDC" TNFa high, IL-6 high | 0.018 | 23.9 |
| cDC TNF- IL-6+ | "cDC" TNFa low, IL-6 high | 1.66 | 6.94 |
| cDC TNF- IL-6­ | "cDC" TNFa low, IL-6 low | 97.3 | 37 |
| cDC TNF+ IL-12­ | "cDC" TNFa high, IL-12 low | 0.98 | 48.4 |
| cDC TNF+ IL-12+ | "cDC" TNFa high, IL-12 high | 0.018 | 7.71 |
| cDC TNF- IL-12+ | "cDC" TNFa low, IL-12 high | 0.32 | 3.5 |
| cDC TNF- IL-12­ | "cDC" TNFa low, IL-12 low | 98.6 | 40.5 |
| cDC TNF+ IFNa ­ | "cDC" TNFa high, IFNa low | 1 | 56 |
| cDC TNF+ IFNa+ | "cDC" TNFa high, IFNa high | 0 | 0.094 |
| cDC TNF- IFNa+ | "cDC" TNFa low, IFNa high | 0.14 | 0.04 |
| cDC TNF- IFNa­ | "cDC" TNFa low, IFNa low | 98.8 | 43.9 |
| pDC TNF+ IL-6 ­ | "pDC" TNFa high, IL-6 low | 0.69 | 59.2 |
| pDC TNF+ IL-6+ | "pDC" TNFa high, IL-6 high | 0.17 | 3.16 |
| pDC TNF- IL-6+ | "pDC" TNFa low, IL-6 high | 0.69 | 0 |
| pDC TNF- IL-6­ | "pDC" TNFa low, IL-6 low | 98.3 | 37.6 |
| pDC TNF+ IL-12­ | "pDC" TNFa high, IL-12 low | 0.86 | 60.2 |
| pDC TNF+ IL-12+ | "pDC" TNFa high, IL-12 high | 0 | 0.6 |
| pDC TNF- IL-12+ | "pDC" TNFa low, IL-12 high | 0.17 | 0.45 |
| pDC TNF- IL-12­ | "pDC" TNFa low, IL-12 low | 98.8 | 38.8 |
| pDC TNF+ IFNa ­ | "pDC" TNFa high, IFNa low | 0.86 | 13.4 |
| pDC TNF+ IFNa+ | "pDC" TNFa high, IFNa high | 0 | 47.4 |
| pDC TNF- IFNa+ | "pDC" TNFa low, IFNa high | 0.34 | 18.5 |
| pDC TNF- IFNa­ | "pDC" TNFa low, IFNa low | 98.6 | 20.8 |

**4.4. Data Transformation Description:**

Data was transformed using FlowJo’s “Define BiExponential Transformation”

function using the above mentioned compensation matrix, with an additional

negative display size set at 0.5 and Positive Decades of “log” Display set at 5.
